# Critical Assessment of Evidence Quality of Meta-Analyses Comparing Sacral 2 Alar–Iliac Fixation with Iliac Screws for Adult Spinal Deformity: An Umbrella Review with Emphasis on Methodological Limitations

**DOI:** 10.3390/jcm15020753

**Published:** 2026-01-16

**Authors:** Ali Haider Bangash, Ananth S. Eleswarapu, Mitchell S. Fourman, Yaroslav Gelfand, Saikiran G. Murthy, Jaime A. Gomez, C. Rory Goodwin, Peter G. Passias, Reza Yassari, Rafael De la Garza Ramos

**Affiliations:** 1 Department of Neurological Surgery, Montefiore Medical Center, Albert Einstein College of Medicine, Bronx, NY 10467, USA; alhaider@montefiore.org (A.H.B.); ygelfand@montefiore.org (Y.G.); samurthy@montefiore.org (S.G.M.); ryassari@montefiore.org (R.Y.); 2 Department of Orthopedic Surgery, Montefiore Medical Center, Albert Einstein College of Medicine, Bronx, NY 10467, USA; aeleswarap@montefiore.org (A.S.E.); mfourman@montefiore.org (M.S.F.); jgomezhe@montefiore.org (J.A.G.); 3 Department of Neurosurgery, Duke University Medical Center, Durham, NC 27710, USA; rory.goodwin@duke.edu (C.R.G.); peter.passias@duke.edu (P.G.P.)

**Keywords:** S2 alar–iliac screw, traditional iliac screw, adult spinal deformity, quality assessment, clinical heterogeneity, primary study overlap

## Abstract

**Background/Objectives**: Adult spinal deformity (ASD) management often requires pelvic fixation, with S2 alar–iliac (S2AI) screws emerging as an alternative to traditional iliac screws. Despite multiple meta-analyses comparing these techniques, the methodological quality of these syntheses and technical heterogeneity across primary studies significantly impact their conclusions and subsequent clinical decision-making. This systematic review evaluates the evidence quality of meta-analyses comparing S2AI with traditional iliac screws for ASD management, focusing on methodological rigor, primary study overlap, and clinical heterogeneity. **Methods**: PubMed, Cochrane, and Epistemonikos were searched for meta-analyses comparing S2AI with iliac screws for patients with ASD. The Quality of Reporting of Meta-analyses (QUOROM) checklist and the revised Assessment of Multiple Systematic Reviews (AMSTAR 2) tool were adopted to assess the methodological quality. Primary study overlap was evaluated using the Corrected Covered Area (CCA) method. Clinical heterogeneity was assessed by examining characteristics of studies included in ≥67% of meta-analyses. **Results**: From a total of 29 publications, six meta-analyses met the inclusion criteria (4807 patients; mean age: 59 years; 33% female). All included meta-analyses exhibited critically low methodological quality per AMSTAR-2, with common flaws including failure to provide lists of excluded studies and lack of a priori protocols. Very high primary study overlap was observed (CCA: 31%), with only 11% (2 of 19) primary studies included in all meta-analyses, whereas 42% (8 of 19) primary studies were included by only a single meta-analysis. Substantial clinical heterogeneity existed regarding patient characteristics, surgical techniques, and outcome definitions. **Conclusions**: This systematic review of meta-analyses identified critically low methodological quality, high primary study overlap, and substantial clinical heterogeneity in the existing evidence comparing pelvic fixation techniques for ASD. While published meta-analyses generally favor S2AI screws, these significant limitations prevent drawing definitive conclusions about superiority. Future research should prioritize high-quality prospective studies with standardized reporting to generate more reliable evidence for improving surgical outcomes in ASD management.

## 1. Introduction

Adult spinal deformity (ASD) management often requires long-segment instrumentation extending to the pelvis for proper correction and stabilization [1]. The evolution of pelvic fixation techniques spans several decades, beginning with Harrington rod extensions in the 1960s, followed by Luque–Galveston techniques in the 1970s and 1980s [2,3]. Iliac screws received recognition as the standard method for pelvic fixation from the 1990s onwards [3]. However, this approach has several limitations, including hardware prominence, wound healing difficulties, and extensive soft tissue dissection required for placement [3]. These challenges prompted the development of alternative fixation techniques.

S2 alar–iliac (S2AI) screws were first described by Sponseller PD and Kebaish KM [4], which offer potential advantages such as placement through the same midline incision, reduced prominence, and better alignment with the rest of the construct [5]. Pelvic fixation, being a critical component of S2AI stabilization, is indicated for long fusions across the lumbosacral junction greater than 3 levels, such as proximal endpoint being above L3 [6]. High-grade spondylolisthesis, three-column osteotomies or vertebral column resections in the low thoracic or lumbar spine, L5-S1 pseudarthrosis, unstable sacral fractures or spinopelvic dissociation are also suggested as indications [6,7]. Furthermore, osteoporosis or poor sacral bone quality, sacral tumors, and pelvic obliquity greater than 15° in neuromuscular scoliosis is also reported as indications for pelvic fixation in adult patients managed with spine surgery [6,8].

As S2AI screws have gained popularity, comparative studies examining outcomes between S2AI and traditional iliac screws have proliferated [9]. However, individual studies often have limited statistical power due to small sample sizes. To address this limitation, several meta-analyses have been conducted to pool available data and provide higher-confidence comparisons between these techniques. Despite these efforts, significant knowledge gaps persist due to methodological variations, primary study overlap, and inconsistent results among existing meta-analyses [10].

This evidence quality gap undermines confidence in clinical recommendations and hinders the identification of genuine research priorities. Therefore, we conducted a comprehensive systematic review of meta-analyses comparing S2AI screws to traditional iliac screws, focusing on three critical dimensions of evidence quality: Methodological rigor using validated assessment tools, quantification of primary study overlap, and exploration of clinical heterogeneity across included studies.

## 2. Materials and Methods

### 2.1. Search Strategy

The systematic review was carried out in accordance with the Preferred Reporting Items for Systematic Review and Meta-Analysis (PRISMA) guidelines [11], with a prospective study protocol (PSP) guiding the objectives, search strategy, and planned analyses developed and subsequently adopted rigorously (Supplementary Materials). Being a systematic review of meta-analyses, ethics approval was exempted from being sought. Trinka AI web application was adopted for the purposes of copyright editing and sentence structure optimization.

The literature search was independently carried out by two investigators (A.H.B. and R.D.G.R.) across PubMed/Medline, Cochrane Database of Systematic Reviews (CDSR), and Epistemonikos, in accordance with the said PSP specifically looking for such systematic reviews with meta-analysis where researchers have tried to establish the efficacy of S2AI by comparing them with traditional iliac screws vis a vis ASD management—by critically appraising and adding together the evidence from primary research studies including randomized controlled trials (RCTs) and prospective as well as retrospective studies. The search strategy comprised the boolean string (“S2 alar-iliac screws” [All Fields] OR “S2AI screws” [All Fields] OR “sacral-alar-iliac” [All Fields] OR “sacral-alar-iliac” [All Fields] OR “S2AI fixation” [All Fields] OR “s2 alar iliac fixation” [All Fields] OR “sacral-2 alar-iliac screws” [All Fields] OR “S2AI technique” [All Fields] OR “s2 alar iliac fixation” [All Fields]) AND (meta-analysis [Filter] OR review [Filter] OR systematicreview [Filter]). The reference lists of the selected, eligible meta-analyses were also independently searched by two investigators (A.H.B. and R.D.G.R.) for relevant primary publications followed by a manual search of those primary publications over Epistemonikos to identify if their evidence has been incorporated in such a meta-analysis that could not be identified during the primary search. The number of studies obtained across each database is reported in the ‘Supplementary Materials’.

### 2.2. Study Endpoints

The primary aim, as determined prior to implementing the search strategy, was the methodological quality assessment of existing meta-analyses comparing S2AI screws to traditional iliac screws for ASD management. The secondary aim was to evaluate the primary study overlap in relevant meta-analyses. The assessment of clinical heterogeneity vis a vis patient characteristics, surgical techniques, and outcome definitions among the most commonly included primary studies was the tertiary aim.

### 2.3. Study Selection

Two investigators (A.H.B. and R.D.G.R.), independently, reviewed the cohort of eligible articles with independent consideration of the abstracts and full-texts. They undertook discussions to reach a consensus in those cases where disagreements arose. The meta-analyses were included if they met the pre-determined inclusion criteria: (1) Adult patients requiring surgical intervention for spinal deformity; (2) a direct comparison of S2AI with traditional iliac screws was made; and (3) systematic review with meta-analysis of primary studies (case series, case–control studies, retrospective cohort studies, prospective cohort studies and/or RCTs) published from the inception of databases until 1 November 2024. Studies were excluded if (1) quantitative meta-analysis was not undertaken; (2) they were any of the primary studies, review articles, conference abstracts, letter to the editor, or any other such correspondence types; and (3) they were drafted in a language other than English. The updated PRISMA flow diagram was adopted to represent the study selection process transparently.

### 2.4. Data Collection and Abstraction

Manual extraction of the required data, in accordance with the pre-determined “Characteristics of studies” table (COST), was carried out independently by two investigators (A.H.B. and R.D.G.R.). The extracted variables included author and year with the number of included studies as well as the number of RCTs included. Moreover, the total number of patients included along with the female population, as well as the respective risk of bias assessment method adopted and respective meta-analysis numerical results were also included. (A.H.B. and R.D.G.R.) authenticated the developed COST. In case of a disagreement, the investigators undertook discussions to reach a consensus.

### 2.5. Quality Assessment and Risk of Bias

Two investigators (A.H.B. and R.D.G.R.), independently, adopted the Quality of Reporting of Meta-analyses (QUOROM) checklist to establish the degree of thorough reporting [12]. The QUOROM checklist provides 18 items covering the abstract, introduction, methods, results, and discussion sections of a meta-analysis report to ensure that all essential information is transparently presented [12]. Moreover, two investigators (A.H.B. and R.D.G.R.), independently, adopted the revised Assessment of Multiple Systematic Reviews (AMSTAR 2) methodology to assess the methodological quality of the included meta-analyses [13]. The AMSTAR 2 tool explores 16 items, including seven critical domains, to provide an overall rating (High, moderate, low, or critically low) of confidence in the results of a systematic review [13]. Both investigators had 100% agreement.

### 2.6. Primary Study Overlap and Clinical Heterogeneity Assessment

Two investigators (A.H.B. and R.D.G.R.), independently, assessed the primary study overlap by exploring the Corrected Covered Area (CCA) measurement [14]. Both investigators had 100% agreement. The CCA is a quantitative method that calculates the percentage of overlap in primary studies, with values ranging from 0% (no overlap) to above 15% (very high overlap). This assessment is based on a citation matrix that maps primary studies included in systematic reviews [14]. For those primary studies that were adopted by at least 67% of the included meta-analyses, the clinical heterogeneity was assessed by exploring the reported patient-related factors [age distribution, sex ratio, body mass index, bone quality (including the presence of a bone quality depleting comorbidity), comorbidities (including diabetes mellitus and cardiovascular disease), severity and type of spinal deformity as well as previous spinal surgeries], and intervention-related factors (screw insertion technique, use of additional fixation methods, intraoperative imaging techniques, and use of navigation systems). Surgeon and hospital-related factors [surgeon’s experience, surgical volume of the hospital, type of hospital (academic vs. community), geographical location], perioperative management (anesthesia protocols, blood loss management strategies, postoperative pain management, and mobilization protocols), and outcome assessment (definition and measurement of primary outcomes, follow-up duration, use of patient-reported outcome measures, and radiographic assessment methods) were also explored.

Moreover, funding and conflicts of interest (funding source and declared conflicts of interest), time-related factors (year of study conduction, changes in surgical techniques or technology over time), study design and methodology (randomization and blinding processes along with sample size power calculations), and other factors were also looked at for such primary studies.

### 2.7. Statistical Analysis

Exploratory data analysis was undertaken, with categorical variables expressed as percentages and continuous variables expressed as means (with standard deviations, where applicable).

## 3. Results

**Figure 1:**
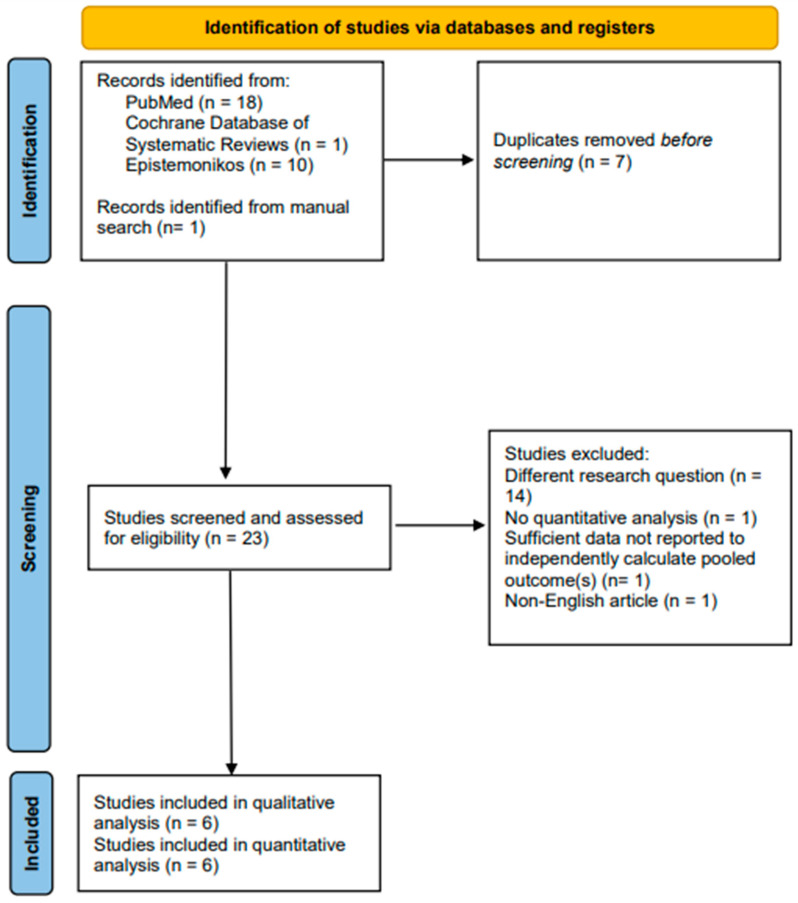
Prisma Flowchart illustrating Search, Selection, and Inclusion of Meta-analyses comparing the Efficacy of S2 Alar–iliac Screws to that of Traditional Iliac Screws for Adult Spinal Deformity Management.

A search carried out through PubMed/Medline, CDSR, and Epistemonikos yielded 29 publications with 1 publication identified via manual search. After removing 7 duplicates, the abstracts and, where required, the full-texts of 23 publications were considered in accordance with the aforementioned inclusion eligibility criteria. Six systematic reviews with meta-analyses satisfied the inclusion eligibility criteria and were, therefore, included in the review (Table 1) [15,16,17,18,19,20]. Those six meta-analyses reviewed 19 primary studies [9,21,22,23,24,25,26,27,28,29,30,31,32,33,34,35,36,37,38]. Concisely stated in the pertinent part of the PRISMA flow diagram are the fundamental reasons why publications were excluded (Figure 1). The list of excluded studies is shared in the ‘Supplementary Materials’.

The included meta-analyses reported on a total of 4807 patients with a mean age of 59 years and 64% female population (n = 1715 of 2666 patients with available sex data) (Table 1). The primary study period covered ranged from 2014 to 2023. 67% (n = 4) meta-analyses adopted PubMed, Cochrane, and Embase for search strategy implementation [15,16,17,18], whereas 67% (n = 4) meta-analyses adopted the Newcastle Ottowa Scale for risk of bias assessment of included primary studies [15,17,18,19]. Only 17% (n = 1) meta-analyses implemented the random-effects model irrespective of the I^2^ statistic value [16]. Moreover, 67% (n = 4) meta-analyses assessed the publication bias affecting the respective meta-analysis results [16,17,18,19]. The included meta-analyses reported that S2AI screws were associated with apparently better postoperative outcomes for ASD when compared with traditional iliac screws (Table 2).

### 3.1. Quality Assessment and Risk of Bias

The QUOROM score ranged from 15 to 16 where none of the included meta-analyses reported methods for validity assessment in sufficient detail to permit replication (Supplementary Table S1). Moreover, none of the meta-analyses reported quality assessment findings in the Abstract ‘Results’ section. The potential biases in the review process (including publication bias) were not discussed sufficiently in 33% (n = 2) meta-analyses [15,18].

Furthermore, all included meta-analyses were found to exhibit ‘critically low’ confidence levels in the results of the respective reviews according to the AMSTAR-2 criteria, as they were found to have more than one critical flaw and were found not to be relied on to provide an accurate and comprehensive summary of the available primary studies (Supplementary Table S1). The list of excluded studies not being provided was found to be the commonest critical flaw found in all included meta-analyses, whereas only a single included meta-analysis reported an apriori establishment of methodology protocol [15]. The most common non-critical flaw was that none of the included meta-analyses explored the sources of funding of the respective included primary studies. Detailed item-by-item quality assessment results for both QUOROM and AMSTAR 2 criteria are presented in Supplementary Table S1.

### 3.2. Primary Study Overlap and Clinical Heterogeneity Assessment

A CCA of 31% was calculated to exist between the primary studies included in the relevant meta-analyses (Supplementary Table S2 details the full citation matrix). A “very high” degree of overlap (>15%) between the primary studies was recognized among the included meta-analyses. Amongst the 19 primary studies that were included by the 6 meta-analyses, 11% (n = 2 of 19) primary studies were included by all meta-analyses [36,37], whereas 42% (n = 8 of 19) primary studies were included by only one of the included meta-analyses [9,21,24,25,26,28,30,32].

The primary studies most commonly included in the meta-analyses (included by 67% of meta-analyses; n = 6 of 19) reported mean age ranging from 58–70 years with the respective female populations ranging from 56–88% (Supplementary Table S3). 67% (n = 4 of 6) most commonly included primary studies did not report body mass index, bone quality, comorbidities, or previous spine surgery history of the respective included patient populations. Degenerative deformity was found to be the most common ailment requiring surgical intervention. However, none of the studies reported the degree of severity of any of the diseases requiring surgical intervention.

83% (n = 5 of 6) most commonly included primary studies did not report the laterality (uni-vs. bilateral screws) in the respective patient population, whereas 50% (n = 3 of 6) most commonly included primary studies did not report the respective screw insertion technique. The studies varied substantially in the reported starting point, exposure, screw direction, guidance, and insertion method of the screws as well as the screw size determination (Supplementary Table S3). Moreover, 50% (n = 3 of 6), 67% (n = 4 of 6), and 83% (n = 5 of 6) most commonly included primary studies, each, did not specify the use of additional fixation methods, intraoperative imaging techniques, and use of navigation systems. Furthermore, Mazur MD et al. adopted an intraoperative stereotactic image guidance system for S2AI screws only, whereas the conventional C-arm was adopted for the IS group [36].

67% (n = 4 of 6) most commonly included primary studies did not report any surgeon and hospital-related factor (surgeon’s experience, surgical volume of the hospital, type of hospital and/or geographical location), whereas none of the most commonly included primary studies reported any measure of perioperative management (anesthesia protocols, blood loss management strategies, postoperative pain management and/or mobilization protocols). The primary studies were found to be considerably variable and deficient in the reporting of definitions of the respective primary as well as secondary outcomes. They also varied substantially in their follow-up durations. Moreover, they also lacked in reporting the respective measurement methods of the outcomes. Moreover, 83% (n = 5 of 6) most commonly included primary studies did not report blinding of any methodology step to reduce bias, whereas none of the most commonly included primary studies adopted sample size power calculations to minimize bias.

Furthermore, although the studies were reported to include data from patients managed between 2001 and 2015, 83% (n = 5 of 6) most commonly included primary studies did not discuss the change in surgical technique over time. 50% (n = 3 of 6) most commonly included primary studies were funded by private organizations, with 33% (n = 2 of 6) studies funded by the same private organization.

## 4. Discussion

ASD significantly impacts the quality of life and often requires pelvic fixation via long-segment instrumentation. Our systematic review critically evaluated the evidence quality of meta-analyses comparing S2AI screws with traditional iliac screws for ASD management through three key dimensions: methodological rigor, primary study overlap, and clinical heterogeneity. We identified pervasive quality concerns, including critically low methodological standards per AMSTAR-2 criteria, very high primary study redundancy (CCA: 31%), and substantial clinical heterogeneity in patient characteristics and surgical techniques. The findings of the included meta-analyses, therefore, warrant cautious interpretation given the underlying quality concerns.

To the best of our knowledge, this study represents the first comprehensive assessment of evidence quality specifically focused on meta-analyses comparing S2AI with iliac screws in ASD management. We adopted a three-dimensional analytical framework integrating methodological quality assessment, quantification of primary study overlap, and detailed examination of clinical heterogeneity. Therefore, it provided a more nuanced understanding than traditional quality assessments that typically focus on methodological criteria alone. This integrated approach revealed that despite apparent consensus across meta-analyses favoring S2AI screws, this consensus may be artificially amplified by redundant analysis of the same primary data and compromised by substantial variations in surgical techniques and patient populations. By quantifying these specific limitations in this important domain of spine surgery, our findings offer spine surgeons a critical lens through which to interpret current evidence and provide researchers with concrete targets for improving future studies. Moreover, the clinical implications of our findings extend beyond simply identifying methodological weaknesses, as they highlight how specific aspects of heterogeneity, such as variations in screw insertion techniques and patient characteristics, may influence the relative performance of these fixation methods in different clinical scenarios.

The methodological quality of all reviewed meta-analyses was critically low according to AMSTAR-2 criteria, consistent with recent findings showing substandard quality in spine surgery meta-analyses [39]. Sathish M et al. reported that 39% of spine surgery meta-analyses from the past two decades had critically low methodological quality [40]. Common critical flaws included failure to analyze conflicts of interest in primary studies, omission of excluded study lists, lack of a priori protocols, and insufficient investigation of publication bias [40,41,42,43]. These methodological deficiencies potentially undermine the validity of current evidence comparing these techniques.

We observed very high primary study overlap (CCA: 31%), indicating significant redundancy that may overestimate precision and certainty in the evidence base. CCA has ranged from 0–73% in spine surgery systematic reviews [39,44]. This redundancy creates a concerning pattern where only 11% of primary studies were included in all meta-analyses [36,37], while 42% of primary studies appeared in only a single relevant meta-analysis [9,21,24,25,26,28,30,32]. This selective inclusion pattern particularly affects outcomes where the repeated analysis of data from frequently included studies potentially amplifies their influence on pooled results, while potentially valuable contradictory evidence from less frequently included studies remains marginalized. With frequently included studies reporting larger effect sizes favoring S2AI screws, their disproportionate representation across meta-analyses might create an artificial consensus that may overstate the true magnitude of benefit, masking underlying uncertainty in the evidence base. Additionally, substantial clinical heterogeneity existed among commonly included studies regarding outcome definitions, surgical techniques, and patient characteristics. Technical variations in screw insertion methods, additional fixation, and intraoperative imaging further limited interpretation. Zhou Z et al. demonstrated that differences in S2AI screw insertion techniques significantly impact biomechanical properties and complication rates [45,46], highlighting how clinical heterogeneity affects outcomes. This heterogeneity may explain discrepancies between studies, such as Eastlack RK et al. finding traditional iliac screws superior to S2AI screws [23], contrary to Ilyas H et al. [37].

The meta-analyses we evaluated consistently reported S2AI screws to lead to significantly improved outcomes when compared with those obtained with traditional iliac screws. However, our systematic review revealed that these conclusions should be interpreted cautiously given the methodological limitations, substantial overlap, and clinical heterogeneity we identified. Recent research has also suggested that these advantages may be less pronounced than initially believed. Nguyen JH et al. reported a relatively low 7.7% complication rate with iliac screws and 91.2% success in lumbosacral fusion [47], highlighting how selective inclusion of primary studies may influence meta-analysis conclusions.

The clinical implications of these evidence gaps are particularly concerning for complex cases where the stakes of fixation failure are highest. Without reliable subgroup analyses accounting for deformity severity, bone quality, and revision status, surgeons lack evidence-based guidance for tailoring fixation strategies to individual patient needs. This may lead to inappropriate technique selection in high-risk patients where fixation failure could necessitate extensive revision surgery with substantial morbidity. We also observed that the existing meta-analyses failed to adequately address evolving surgical approaches in complex ASD cases. Increasingly, complex deformity surgeons are utilizing triple or quad rod constructs for enhanced stability in ASD correction [48]. In these advanced constructs, direct iliac screws may be used in conjunction with S2AI screws rather than as competing alternatives. This combined approach potentially leverages the biomechanical advantages of both fixation methods to address the substantial forces at the lumbosacral junction in severe deformity cases [49]. This paradigm shift from viewing these techniques as competing alternatives to complementary tools represents a solution to the limitations of each technique and offers a more nuanced approach to complex deformity correction. The absence of this perspective in contemporary meta-analyses creates a false dichotomy that may inappropriately simplify clinical decision-making and limit innovation in surgical technique. The complementary use of both fixation techniques in multi-rod constructs represents an important consideration not captured in the comparative meta-analyses that met the inclusion criteria.

Future meta-analyses should clearly outline their unique contributions, rigorously follow established protocols like PRISMA and AMSTAR 2 [11,13], as well as be held to higher standards by journal editors and reviewers. Developing a unified database of primary spine studies could reduce duplication, while standardized reporting of patient characteristics, surgical techniques, and outcomes would reduce clinical heterogeneity. Implementation of core outcome sets for ASD surgery would enhance comparability, and advanced subgroup analyses and meta-regression approaches could better account for clinical heterogeneity. Future research should also consider conducting sensitivity analyses on high-quality meta-analyses comparing these techniques, when they become available, to verify the robustness of conclusions across various methodological assumptions. Global ASD surgery registries could provide empirical data on how these variations affect clinical practice.

Additional research should also explore stratification by key patient and surgical characteristics, including guidance devices and screw insertion techniques [45,46,50]. Standardized training programs and learning curve analyses for S2AI screw placement could optimize technique efficacy across surgical settings [51]. Further investigation is also needed regarding patient-specific risk factors such as diabetes mellitus that could impact the risk of complications with S2AI screws [34]. Furthermore, the long-term effects of reduced complications on patient outcomes and healthcare costs remain unclear, necessitating comprehensive comparative cost-effectiveness assessments.

The key strengths of our study include a comprehensive literature search methodology, rigorous methodological quality assessment, and detailed evaluation of clinical heterogeneity across surgical techniques and patient populations. Limitations include the relatively small body of high-quality comparative studies available in the literature and the inability to account for surgeon experience as well as technical proficiency, which could potentially influence outcomes with these technically demanding procedures. Despite these limitations, this systematic review provides a critical assessment of the contemporary evidence regarding spinopelvic fixation options in ASD management and identifies important considerations for surgical decision-making and future research.

## 5. Conclusions

Our systematic review of meta-analyses identified critically low methodological quality, high primary study overlap, and substantial clinical heterogeneity in the existing evidence comparing S2AI with traditional iliac screws for ASD management. While the included meta-analyses consistently report advantages for S2AI screws, these findings must be interpreted with extreme caution given the significant methodological limitations we identified. The apparent consensus may be artificially amplified by redundant analysis of the same primary data and compromised by substantial variations in surgical techniques and patient populations. Given these evidence limitations, clinical decision-making should incorporate patient-specific factors, surgeon experience, and institutional preferences while recognizing that neither technique has been conclusively demonstrated to be superior across all clinical scenarios. Future research should focus on high-quality prospective studies with standardized reporting of patient characteristics, surgical techniques, and outcomes to generate more reliable evidence and improve surgical outcomes for patients with ASD.

## Figures and Tables

**Table 1:** Study Particulars and Demographics of Systematic Reviews with Meta-analyses exploring Reoperation and Wound-related Complication Rates after S2 Alar–iliac Screws compared to those after Traditional Iliac Screws for Adult Spinal Deformity Management.

Author and Year	Number of Included Studies	Study Period Covered	Databases Searched	Risk of Bias Assessment Method	Total Number of Patients	Age	% Female
Rahmani R et al., 2024 [15]	15	2015–2022	PubMed. Cochrane and Embase	Newcastle Ottawa scale (NOS)	1502	64.63 years	71% (n = 816 of 1151 patients) (data reported for 14 studies)
Shin HK et al., 2023 [16]	10	2015–2023	PubMed. Cochrane, Embase and Web of Science	The Risk of Bias In Nonrandomized Studies of Interventions (ROBINS-I) tool	1931	62.26 years (data reported for 7 studies)	73% (n = 283 of 389 patients) (data reported for 5 studies)
Gao Z et al., 2021 [17]	8	2014–2019	PubMed. Cochrane and Embase	NOS	426	40.1 years	69% (n = 295)
Hasan MY et al., 2020 [18]	3 *	2015	PubMed. Cochrane, Embase, Web of Science and Scopus	NOS	218 *	62.3 years *	Not reported
Keorochana G et al., 2019 [19]	7 *	2015–2019	PubMed and Scopus	NOS	422 *	63.58 years *	31% (n = 129)
De la Garza Ramos R et al., 2018 [20]	5	2015–2017	PubMed	Unspecified scale	323	62.46 years (data reported for 4 studies)	69% (n = 192 of 278 patients) (data reported for 4 studies)

* Pertinent to adult patient population.

**Table 2:** Meta-analytical Results and Inferred Direction of Findings of Systematic Reviews with Meta-analyses exploring Reoperation and Wound-related Complication Rates after S2 Alar–iliac Screws compared to those after Traditional Iliac Screws for Adult Spinal Deformity Management.

Author and Year	Meta-Analysis Results	Direction of Findings
Rahmani R et al., 2024 [15]	-Reoperation odds (of the iliac screws) [(n = 1113 patients) Odds ratio (OR) = 2.45 (95% CI: 1.25–4.77) (I^2^ = 57%)] -Wound complications odds (of iliac screws) [(n = 699 patients) OR = 5.94 (95% CI: 1.55–22.79) (I^2^ = 77%)] -Wound infection odds (of iliac screws) [(n = 622 patients) OR = 7.4 (95% CI: 2.12–25.85) (I^2^ = 55%)] -Wound dehiscence odds (of iliac screws) [(n = 670 patients) OR = 4.47 (95% CI: 2.11–9.47) (I^2^ = 49%)]	S2AI screws have reportedly lower complication rates (wound complications, screw prominence, and revision surgeries) than traditional iliac screws in adult spinopelvic deformity (ASD) surgeries.
Shin HK et al., 2023 [16]	-Proportion of revision [(n = 1642 patients) Significantly higher in the Iliac screws group (21%; 95% CI, 11.8–30.2%) than that in the S2AI group (8.5%; 95% CI, 3.4–13.6%) (*p* = 0.02)] -Proportion of wound complications [(n = 389 patients) Significantly higher in the Iliac screws group (31.7%; 95% CI, 11.5–51.9%) than that in the S2AI group (3.9%; 95% CI, ZERO–7.9%) (*p* < 0.01)]	While S2AI and iliac screws showed comparable implant failure rates, S2AI screws were associated with reportedly lower revision rates, fewer screw prominence issues, and reduced wound complications.
Gao Z et al., 2021 [17]	-Reoperation odds (of S2AI screws) [(n = 239 patients) OR = 0.2 (95% CI: 0.09–0.44) (I^2^ = ZERO%)] -Wound infection odds (of S2AI screws) [(n = 157 patients) OR = 0.16 (95% CI: 0.04–0.58) (I^2^ = ZERO%)]	S2AI screws reportedly demonstrated comparable effectiveness to traditional iliac screws in managing spinal alignment parameters while being associated with reportedly lower rates of hardware complications (implant failure, screw loosening, prominence), reoperation, and wound infection. A non-significant trend toward improved pain management and reduced blood loss with S2AI screws was also reported.
Hasan MY et al., 2020 [18]	-Revision (of S2AI screws) [(n = 218 patients) OR = 7.84 (95% CI: 3.22–19.08) (I^2^ = 0%)]	In adult patients, S2AI screws are reportedly associated with significantly lower revision surgery rates compared to traditional iliac screws.
Keorochana G et al., 2019 [19]	-Complications (of S2AI screws) [(n = 257 patients) Relative risk (RR) = 2.32 (95% CI: 1.60–3.38) (I^2^ = 9%)] -Revisions (of S2AI screws) [(n = 322 patients) RR = 1.94 (95% CI: 1–3.73) (I^2^ = 52.9%)]	Adult patients with iliac screws had reportedly a higher risk of complications and revisions compared to those with S2AI screws.
De la Garza Ramos R et al., 2018 [20]	-Requirement of revision surgery (in S2AI group) [(n = 323 patients) OR = 0.32 (95% CI: 0.18–0.58) (I^2^ = 74%)] -Wound infection odds (in S2AI group) [(n = 278 patients) OR = 0.09 (95% CI: 0.03–0.26) (I^2^ = 50%)]	S2AI screws were associated with reportedly lower revision surgery requirements, reduced wound infection rates, and decreased screw prominence compared to traditional iliac screws.

OR = Odds ratio; S2AI = S2 alar–iliac; ASD = Adult spinopelvic deformity; RR = Relative risk.

## Data Availability

The original contributions presented in this study are included in the article and Supplementary Material. Further inquiries can be directed to the corresponding author.

## Supplementary Materials

The following supporting information can be downloaded at https://www.mdpi.com/article/10.3390/jcm15020753/s1, Prospective Study Protocol & Search Strategy Implementation; QUOROM and AMSTAR 2 scoring sheet; Primary study citation matrix used for CCA calculations; Detailed clinical heterogeneity assessment data; and PRISMA checklist. Supplementary Table S1. Quality of Reporting of Meta-analyses (QUROM) and Revised Assessment of Multiple Systematic Reviews (AMSTAR 2) Analyses with detailed specifics for Systematic Reviews with Meta-analyses exploring Reoperation and Wound-related Complication Rates after S2 Alar-iliac Screws compared to those after Traditional Iliac Screws for Adult Spinal Deformity Management. Supplementary Table S2. Citation Matrix of Primary Studies adopted in the Meta-analyses exploring Reoperation and Wound-related Complication Rates after S2 Alar-iliac Screws compared to those after Traditional Iliac Screws for Adult Spinal Deformity Management. Supplementary Table S3. Evaluation of Clinical Heterogeneity in Primary Studies adopted by 67% of the Meta-analyses exploring Reoperation and Wound-related Complication Rates after S2 Alar-iliac Screws compared to those after Traditional Iliac Screws for Adult Spinal Deformity Management.

## Abbreviations

The following abbreviations are used in this manuscript:

ASD	Adult spinal deformity
S2AI	S2 alar–iliac
PRISMA	Preferred Reporting Items for Systematic Review and Meta-Analysis
QUOROM	Quality of Reporting of Meta-analyses
AMSTAR 2	Assessment of Multiple Systematic Reviews
CCA	Corrected Covered Area
