# Supplementary material for: Critical Assessment of Evidence Quality of Meta-Analyses Comparing Sacral 2 Alar–Iliac Fixation with Iliac Screws for Adult Spinal Deformity: An Umbrella Review with Emphasis on Methodological Limitations
Source: J Clin Med. 2026 Jan 16;15(2):753. doi: 10.3390/jcm15020753 (PMC12842343; doi:10.3390/jcm15020753)
Supplement: Supplementary file 1 [file jcm-15-00753-s001.zip › jcm-4038047-supplementary.pdf]

Supplementary materials

Prospective Study Protocol & Search Strategy Implementation

- **P**atients
  - Adult patients suffering from spinal deformity
  - Exclusion:
    - Pediatric patients suffering from spinal deformity
- **I**ntervention
  - Sacral-2-alar-iliac (S2AI) screws
- **C**omparator
  - Traditional iliac screws
    - Exclusion:
      - Galvestion rods
- **O**utcome
  - Methodological quality assessment
    - QUOROM checklist
    - AMSTAR-2 analysis
    - Primary study overlap assessment via Corrected covered area assessment
  - Clinical heterogeneity assessment of studies included by 67% of meta-analyses
- **S**tudy design
  - Inclusion:

        - Systematic review with meta-analysis
    - Exclusion:
        - Literature reviews
        - Randomized controlled clinical trials
        - Non-randomized clinical trials
        - Preclinical studies
        - Cohort studies
        - Case-control studies
        - Case series
        - Case reports
        - Letter to editor, commentaries and all other such types of correspondences

MeSH terms were acquired for aforementioned relevant terms from the MeSH resource of NCBI.

Search strategy implemented across all 3 databases on: November 1, 2024

- **PubMed**
    - "S2 alar-iliac screws"[All Fields] OR "S2AI screws"[All Fields] OR "sacral-alar-iliac"[All Fields] OR "sacral-alar-iliac"[All Fields] OR "S2AI fixation"[All Fields] OR "s2 alar iliac fixation"[All Fields] OR "sacral-2 alar-iliac screws"[All Fields] OR "S2AI technique"[All Fields] OR "s2 alar iliac fixation"[All Fields] 218
        - **(meta-analysis[Filter] OR review[Filter] OR systematicreview[Filter]) 18**
- **CSDR**
    - S2 alar-iliac screws 2
    - S2AI screws 4
    - S2AI fixation 6
    - s2 alar iliac fixation 3
    - sacral-2 alar-iliac screws 2
    - S2AI technique 3
    - s2 alar iliac fixation 3
    - sacral alar iliac 5
    - sacral-alar-iliac 1
    - #1 OR #2 OR #3 OR #4 OR #5 OR #6 OR #7 OR #8 OR #9 9
        - **Filter: Cochrane review 1**
- **Epistemonikos**

- (title:(S2 alar-iliac screws) OR abstract:(S2 alar-iliac screws)) OR (title:(S2AI screws) OR abstract:(S2AI screws)) OR (title:(S2AI fixation) OR abstract:(S2AI fixation)) OR (title:(s2 alar iliac fixation) OR abstract:(s2 alar iliac fixation)) OR (title:(sacral-2 alar-iliac screws) OR abstract:(sacral-2 alar-iliac screws)) OR (title:(S2AI technique) OR abstract:(S2AI technique)) OR (title:(s2 alar iliac fixation) OR abstract:(s2 alar iliac fixation)) 22
    - **Filter: Systematic review 10**

- Total articles: 29
    - Duplicates removed: 7
        - Total articles screened: 22
            - **Articles included from screening: 5**
- **Articles excluded from screening (n= 22)**
    - **Different research question (n= 14)**
        - Cheuk DK, Wong V, Wraige E, Baxter P, Cole A. Surgery for scoliosis in Duchenne muscular dystrophy. Cochrane Database Syst Rev. 2015;2015(10):CD005375. Published 2015 Oct 1. doi:10.1002/14651858.CD005375.pub4
        - Gang L, Qiang Y, Yonghong H, Shucai D. Advantages and significance of S2 alar iliac screw fixation in sacropelvic fixation. Chin J Tissue Eng Res. 2021;25(21):3395. doi:10.3969/j.issn.2095-4344.3861

- Khalifeh K, Brown NJ, Pennington Z, Pham MH. Spinal Robotics in Adult Spinal Deformity Surgery: A Systematic Review. Neurospine. 2024;21(1):20-29. doi:10.14245/ns.2347138.569
- Cirrincione P, Widmann RF, Heyer JH. Advances in robotics and pediatric spine surgery. Curr Opin Pediatr. 2023;35(1):102-109. doi:10.1097/MOP.0000000000001199
- Kanno H, Onoda Y, Hashimoto K, Aizawa T, Ozawa H. Innovation of Surgical Techniques for Screw Fixation in Patients with Osteoporotic Spine. J Clin Med. 2022;11(9):2577. Published 2022 May 4. doi:10.3390/jcm11092577
- Ravindra VM, Mazur MD, Brockmeyer DL, et al. Clinical Effectiveness of S2-Alar Iliac Screws in Spinopelvic Fixation in Pediatric Neuromuscular Scoliosis: Systematic Literature Review. Global Spine J. 2020;10(8):1066-1074. doi:10.1177/2192568219899658
- Casaroli G, Bassani T, Brayda-Bruno M, Luca A, Galbusera F. What do we know about the biomechanics of the sacroiliac joint and of sacropelvic fixation? A literature review. Med Eng Phys. 2020;76:1-12. doi:10.1016/j.medengphy.2019.10.009
- El Dafrawy MH, Raad M, Okafor L, Kebaish KM. Sacropelvic Fixation: A Comprehensive Review. Spine Deform. 2019;7(4):509-516. doi:10.1016/j.jspd.2018.11.009
- Katsuura Y, Chang E, Sabri SA, Gardner WE, Doty JF. Anatomic Parameters for Instrumentation of the Sacrum and Pelvis: A Systematic

Review of the Literature. J Am Acad Orthop Surg Glob Res Rev. 2018;2(8):e034. Published 2018 Aug 2. doi:10.5435/JAAOSGlobal-D-18-00034

- Laratta JL, Shillingford JN, Meredith JS, Lenke LG, Lehman RA, Gum JL. Robotic versus freehand S2 alar iliac fixation: in-depth technical considerations. J Spine Surg. 2018;4(3):638-644. doi:10.21037/jss.2018.06.13
- Jain A, Hassanzadeh H, Strike SA, Menga EN, Sponseller PD, Kebaish KM. Pelvic Fixation in Adult and Pediatric Spine Surgery: Historical Perspective, Indications, and Techniques: AAOS Exhibit Selection. J Bone Joint Surg Am. 2015;97(18):1521-1528. doi:10.2106/JBJS.O.00576
- Mattei TA, Fassett DR. Combined S-1 and S-2 sacral alar-iliac screws as a salvage technique for pelvic fixation after pseudarthrosis and lumbosacropelvic instability: technical note. J Neurosurg Spine. 2013;19(3):321-330. doi:10.3171/2013.5.SPINE121118
- Mattei TA, Fassett DR. Low-profile pelvic fixation with sacral alar-iliac screws. Acta Neurochir (Wien). 2013;155(2):293-297. doi:10.1007/s00701-012-1560-5
- Shen FH, Mason JR, Shimer AL, Arlet VM. Pelvic fixation for adult scoliosis. Eur Spine J. 2013;22 Suppl 2(Suppl 2):S265-S275. doi:10.1007/s00586-012-2525-3

- **No quantitative analysis (n= 1)**

    - Hirase T, Shin C, Ling J, et al. S2 alar-iliac screw versus traditional iliac screw for spinopelvic fixation: a systematic review of comparative biomechanical studies. Spine Deform. 2022;10(6):1279-1288. doi:10.1007/s43390-022-00528-2
  - **Sufficient data not reported to independently calculate pooled outcome(s) (n= 1)**
    - Zhao J, Nie Z, Zhou J, Liao D, Liu D. Incidence and Risk Factors of the Caudal Screw Loosening after Pelvic Fixation for Adult Spinal Deformity: A Systematic Review and Meta-analysis. Asian Spine J. 2024;18(1):137-145. doi:10.31616/asj.2022.0421
  - **Non-English article (n= 1)**
    - Zhang N, Yu B. Zhongguo Xiu Fu Chong Jian Wai Ke Za Zhi. 2018;32(6):764-768. doi:10.7507/1002-1892.201711124

- **Articles included from manual exploration: 1**
  - Shin HK, Park JH, Jeon SR, et al. Sacropelvic Fixation for Adult Deformity Surgery Comparing Iliac Screw and Sacral 2 Alar-Iliac Screw Fixation: Systematic Review and Updated Meta-Analysis. Neurospine. 2023;20(4):1469-1476. doi:10.14245/ns.2346654.327

Supplementary Tables

Supplementary Table 1. Quality of Reporting of Meta-analyses (QUROM) and Revised Assessment of Multiple Systematic Reviews (AMSTAR 2) Analyses with detailed specifics for Systematic Reviews with Meta-analyses exploring Reoperation and Wound-related Complication Rates after S2 Alar-iliac Screws compared to those after Traditional Iliac Screws for Adult Spinal Deformity Management

	Included studies					
	Rahmani R et al., 2024 [15]	Shin HK et al., 2023 [16]	Gao Z et al., 2021[17]	Hasan MY et al., 2020 [18]	Keorochana G et al., 2019 [19]	De la Garza Ramos R et al., 2018 [20]
**Comprehensiveness of Reporting**						
QUOROM score	15(The ‘Methods’ section of Abstract does not detail methods for validity assessment and quantitative data synthesis in sufficient detail to permit replication. Excluded studies and qualitative	16(The ‘Methods’ section of Abstract does not detail methods for validity assessment in sufficient detail to permit replication. Excluded studies and qualitative findings not reported in the	15(The title does not identify the report as a meta-analysis or systematic review. The ‘Methods’ section of Abstract does not detail methods for validity assessment and quantitative data synthesis in	15(The ‘Methods’ section of Abstract does not detail methods for validity assessment, study characteristics, and quantitative data synthesis in sufficient detail to permit replication. The ‘Results’ section	15(The ‘Methods’ section of Abstract does not detail methods for validity assessment, study characteristics, and quantitative data synthesis in sufficient detail to permit replication. The ‘Results’ section	15(The abstract does not detail the methods for validity assessment and data abstraction sufficiently for replication & excluded studies as well as qualitative findings not indicated in the ‘Results’ section of the Abstract.

	findings not reported in the 'Results' section of the Abstract. Potential biases in the review process (including publication bias) were not discussed sufficiently.)	'Results' section of the Abstract.)	sufficient detail to permit replication. Excluded studies and qualitative findings not reported in the 'Results' section of the Abstract)	of Abstract does not detail the qualitative findings. Potential biases in the review process (including publication bias) and future research agenda were not discussed sufficiently.)	of Abstract does not detail the qualitative findings. Restrictions in studies searching including years considered, publication status and/or the language of publication were not reported.)	Moreover, the process or processes used for data abstraction (e.g., completed independently, in duplicate) not provided.)
**Internal Validity (Methodological Quality)**<br>AMSTAR 2 appraised confidence in the results of the review	Critically Low (The review has more than one critical flaw and should not be relied on to provide an accurate and comprehensive summary of the available studies. Critical flaws: List of Excluded Studies not provided & No specific Investigation of	Critically Low (The review has more than one critical flaw and should not be relied on to provide an accurate and comprehensive summary of the available studies. Critical flaws: No apriori establishment of protocol & the List of Excluded Studies not	Critically Low (The review has more than one critical flaw and should not be relied on to provide an accurate and comprehensive summary of the available studies. Critical flaws: No apriori establishment of protocol & the List of Excluded Studies not	Critically Low (The review has more than one critical flaw and should not be relied on to provide an accurate and comprehensive summary of the available studies. Critical flaws: No apriori establishment of protocol, the List of Excluded Studies with justification not	Critically Low (The review has more than one critical flaw and should not be relied on to provide an accurate and comprehensive summary of the available studies. Critical flaws: No apriori establishment of protocol, the List of Excluded Studies with justification not	Critically Low (The review has more than one critical flaw and should not be relied on to provide an accurate and comprehensive summary of the available studies. Critical flaws: No apriori establishment of protocol, the List of Excluded Studies with

	Publication Bias. Non-critical flaw: No exploration of Sources of Funding for Included Studies.)	provided. Non-critical flaw: No exploration of Sources of Funding for Included Studies.)	provided. Non-critical flaw: No exploration of Sources of Funding for Included Studies.)	provided. Non-critical flaw: No exploration of Sources of Funding for Included Studies)	provided. Non-critical flaw: No exploration of Sources of Funding for Included Studies)	justification not provided & publication bias not explored. Non-critical flaws: Non-duplication study selection and data extraction & no exploration of Sources of Funding for Included Studies.)

Supplementary Table 2. Citation Matrix of Primary Studies adopted in the Meta-analyses exploring Reoperation and Wound-related Complication Rates after S2 Alar-iliac Screws compared to those after Traditional Iliac Screws for Adult Spinal Deformity Management

**Primary publications**	**Meta-analyses**					
	Rahmani R et al., 2024 [15]	Shin HK et al., 2023 [16]	Gao Z et al., 2021 [17]	Hasan MY et al., 2020 [18]	Keorochana G et al., 2019 [19]	De la Garza Ramos R et al., 2018 [20]
von Glinski A et al., 2022 [21]	✅					
Lee NJ et al., 2023 [22]	✅	✅				
Eastlack RK et al., 2022 [23]	✅	✅				
Martin CT et al., 2023 [24]		✅				

McDonnell JM et al., 2022 [25]		✅					
Park GO et al., 2021 [26]	✅						
Luo Q et al., 2021 [27]	✅	✅					
Krieg SM et al., 2021 [9]	✅						
Lee KY et al., 2020 [28]	✅						
Ishida W et al., 2019 [29]	✅				✅		
Li J et al., 2019 [30]			✅				
Nazemi AK et al., 2018 [31]	✅	✅	✅		✅		

Liu Z et al., 2018 [32]			✅			
Ishida W et al., 2017 [33]	✅		✅		✅	✅
Elder BD et al., 2017 [34]	✅	✅		✅	✅	✅
Ishida W et al., 2016 [35]	✅		✅			
Mazur MD et al., 2015 [36]	✅	✅	✅	✅	✅	✅
Ilyas H et al., 2015 [37]	✅	✅	✅	✅	✅	✅
Guler UO et al., 2015 [38]	✅	✅	✅		✅	✅

Supplementary Table 3. Evaluation of Clinical Heterogeneity in Primary Studies adopted by 67% of the Meta-analyses exploring Reoperation and Wound-related Complication Rates after S2 Alar-iliac Screws compared to those after Traditional Iliac Screws for Adult Spinal Deformity Management

Primary studies	Measures of Clinical Heterogeneity
Guler UO et al., 2015 [38]	–**Patient-related factors:**<br>-**Mean Age:** 63 ± 14 years<br>-**Sex ratio:** 88% female<br>-**Body mass index (BMI):** Not provided<br>-**Bone quality:** Not specified<br>-**Comorbidities:** Not specifically listed<br>-**Severity and type of spinal deformity:** Degenerative (44.4%), failed back (24.4%), and other conditions (31%) with undefined severity<br>-**Previous spinal surgeries:** Not reported<br><br>–**Intervention-related factors:**<br>-**Laterality:** Not reported<br>-**Surgical screw insertion technique:**<br>● **Starting point, Exposure, Screw direction, and Guidance method:** Not specified for S2 Alar-iliac (S2AI) and traditional iliac screws (IS)<br>● **Screw size and material:** 5.5 mm Ti alloy rods<br>-**Use of additional fixation methods:** Not specified<br>-**Intraoperative imaging techniques:** Not mentioned<br>-**Use of navigation systems:** Not reported<br><br>–**Surgeon and hospital-related factors:**<br>-**Surgeon's experience:** Not mentioned<br>-**Surgical volume of the hospital:** Not provided<br>-**Type of hospital (academic vs. community):** Not specified

	**-Geographical location:** European Spine Study Group centers with the specific location not provided<br><br>**–Perioperative management:**<br>**-Anesthesia protocols:** Not discussed<br>**-Blood loss management strategies:** Not mentioned<br>**-Postoperative pain management:** Not detailed<br>**-Mobilization protocols:** Not provided<br><br>**–Outcome assessment:**<br>**-Definition and measurement of primary outcomes:**<br>● The following outcome was defined:<br>○ **Global tilt:** The angle between the line drawn from the center of C7 to the center of the sacral endplate and the line drawn from the center of the sacral endplate to the center of femoral heads; is equal to the arithmetic sum of pelvic tilt and T1 sagittal tilt<br>○ **Lordosis gap:** The posterior angle between the line connecting the centroid of T1 to the center of the upper end plate of the sacrum and the line connecting the center of the femoral heads to the center of the upper endplate of the sacrum, hence, the arithmetic sum of T1 sagittal tilt and pelvic tilt<br>● Mechanical failure, sagittal vertical axis (SVA), T1 sagittal tilt, and health-related quality of life (HRQoL) parameters were not defined.<br>● The undefined HRQoL parameters were measured by the Oswestry disability index (ODI), 36-item short-form physical component and mental component health surveys (SF-36; PCS and MCS), and the Scoliosis Research Society 22-item patient questionnaire (SRS-22). The measurement method of none of the other outcomes was reported.<br>**-The average follow-up** was 17.6 months (median 24 months).<br>**-Use of patient-reported outcome measures:** ODI, SF-36- PCS, and MCS, as well as SRS-22 were adopted to assess undefined HRQoL parameters.<br>**-Radiographic assessment methods:** Not reported<br><br>**–Study design and methodology:**<br>-Prospective, multicentric database<br>-Non-randomized study with no blinding procedures<br>**-Sample size and power calculations:** Not reported

	**–Time-related factors:**<br>**-Year of study conduction:** Not specified<br>**-Changes in surgical techniques or technology over time:** Not discussed in detail<br><br>**–Funding and conflicts of interest:**<br>**-Source of funding:** Sponsored by Depuy Spine Inc. (a subsidiary of a private conglomerate)<br>**-Declared conflicts of interest:** Declared to be none
Ilyas H et al., 2015 [37]	**–Patient-related factors:**<br>**-Mean Age:** 66.3 years (S2AI), 64.3 years (IS)<br>**-Sex Ratio:** 63.6% female (S2AI), 79.1% female (IS)<br>**-BMI:** Not mentioned<br>**-Bone quality:** Patient specifics not provided<br>**-Comorbidities:** Not specified<br>**-Severity and type of spinal deformity:** Unreported<br>**-Previous spinal surgeries:** Not specified<br><br>**–Intervention-related factors:**<br>**-Laterality:** Not reported<br>**-Surgical screw insertion technique:**<br>● **S2AI:**<br>○ **Starting point:** 'Recommended' starting site (specific location not reported)<br>○ **Exposure:** Lateral dissection from the midline to just past the S1 and S2 foramina<br>○ **Screw direction:** Across the sacroiliac joint (SIJ), then directed into the inferior aspect of the iliac teardrop<br>○ **Guidance:** Fluoroscopy<br>○ **Insertion method:** Using a probe<br>○ **Screw size:** 8 mm in diameter × 80 mm in length<br>● **IS:**<br>○ **Starting point:** Posterior superior iliac spine (PSIS)<br>○ **Exposure:** Frequently placed through a separate fascial incision, occasionally by dissecting laterally up and over the iliac crest. A notch is created to reduce screw prominence.<br>○ **Screw direction:** Trajectory directed over the top of the acetabulum and just above the sciatic notch<br>○ **Guidance:** Fluoroscopy

	○ **Insertion method:** Unreported<br>○ **Screw size:** Unspecified<br>**-Use of additional fixation methods:** Not specified<br>**-Intraoperative imaging techniques:** Fluoroscopy used to confirm instrumentation positioning for both: S2AI and IS<br>**-Use of navigation systems:** Not specified<br><br>**–Surgeon and hospital-related factors:**<br>**-Surgeon's experience:** Not specified<br>**-Surgical volume of the hospital:** Not specified<br>**-Type of hospital:** Not mentioned, but the study was conducted at Saint Louis University School of Medicine, suggesting an academic setting<br>**-Geographical location:** Saint Louis, Missouri, USA<br><br>**–Perioperative management:**<br>**-Anesthesia protocols:** Not specified<br>**-Blood loss management strategies:** Not specified<br>**-Postoperative pain management:** Not specified<br>**-Mobilization protocols:** Not specified<br><br>**–Outcome assessment:**<br>**-Definition and measurement of primary outcomes:**<br>● The following outcome was defined:<br>○ **Acute infection:** An infection in the surgical site within 3 months of surgery<br>○ **Delayed infection:** An infection with clinical presentation after 3 months post-operatively<br>○ **Late pain:** Pain over the gluteal region present after at least 3 months postoperatively<br>● Average blood loss, dehiscence, total loosening, average time of loosening, implant breakage, average time of implant breakage, revision surgery, average time of revision surgery, average time of late pain onset, and implant-related complications were not defined.<br>● The measurement method of none of the outcomes was reported.<br>**-Follow-up duration:** 22.3 months (S2AI), 29.6 months (IS)<br>**-Use of patient-reported outcome measures:** Not reported<br>**-Radiographic assessment methods:** Radiographs reviewed for implant loosening or breakage only with no calculations or measurements performed

	–**Study design and methodology:**<br>-Retrospective, non-randomised study with no blinding procedures<br>**Sample size and power calculations:** Not specified<br><br>–**Time-related factors:**<br>**-Year of study conduction:** Adult surgeries were performed between 2001 and 2011<br>**-Changes in surgical techniques or technology over time:** Not discussed<br><br>–**Funding and conflicts of interest:**<br>**-Source of funding:** Not explicitly mentioned<br>**-Declared conflicts of interest:** Declared to be none
Mazur MD et al., 2015 [36]	–**Patient-related factors:**<br>**-Mean age:** 58 ± 14 years (S2AI), 64 ± 11 years (IS)<br>**-Sex ratio:** 57% female (S2AI), 76% female (IS)<br>**-BMI:** Not mentioned<br>**-Bone quality:** Not specified<br>**-Comorbidities:** Not provided<br>**-Severity and type of spinal deformity:** Scoliosis or degenerative disease [48% (S2AI), 65% (IS)], failed lumbar fusion [35% (S2AI), 32% (IS)], infection or tumor [17% (S2AI), 3% (IS)] with undefined severity and undetermined statistical significance of rate difference<br>**-Previous spinal surgeries:** Previous lumbar fusion surgery [48% (S2AI), 38% (IS)] with undetermined statistical significance of rate difference<br><br>–**Intervention-related factors:**<br>**-Laterality:** Not reported<br>**-Surgical screw insertion technique:**<br>● **S2AI**<br>○ **Starting point:** 5 mm caudal and 2–3 mm lateral to the S-1 foramen<br>○ **Exposure:** Limited subperiosteal dissection on the sacrum to expose entry points<br>○ **Screw direction:** Angled toward the greater trochanter, rostral to the sciatic notch<br>○ **Guidance:** Stereotactic image guidance (O-arm Surgical Imaging System/StealthStation)

	○ **Screw size:** Commonly 8.0–8.5 mm in diameter and 80–100 mm in length<br>○ Trajectory enabled the screw to cross the cortical surfaces of the SIJ and engage the dense bone above the sciatic notch; the screw head was aligned with rostral instrumentation without connector devices<br>● **IS**<br>○ **Starting point:** 1 cm rostral to the inferior end of the PSIS and 1 cm deep to its superficial ridge<br>○ **Exposure:** Subperiosteal dissection to the PSIS<br>○ **Screw direction:** Angled toward the sciatic notch<br>○ **Guidance:** Fluoroscopic guidance using C-arm with tear drop and lateral views<br>○ **Screw size:** Typically 7.5–9.0 mm in diameter and 80–100 mm in length<br>○ Polyaxial pelvic screw heads were buried deep to the superficial ridge of the PSIS; connector devices were used to link iliac bolts to rostral instrumentation<br>**-Use of additional fixation methods:** bone morphogenetic protein (BMP) [52% (S2AI), 92% (IS)] with undetermined statistical significance of rate difference<br>**-Intraoperative imaging techniques:** Stereotactic image guidance (O-arm Surgical Imaging System/StealthStation) for S2AI, Fluoroscopic guidance using C-arm with tear drop and lateral views for IS<br>**-Use of navigation systems:** For S2AI screws insertion only<br><br>**–Surgeon and hospital-related factors:**<br>**-Surgeon's experience:** Not detailed<br>**-Surgical volume of the hospital:** Not provided<br>**-Type of hospital (academic vs. community):** Not reported<br>**-Geographical location:** Unspecified<br><br>**–Perioperative Management:**<br>**-Anesthesia protocols:** Not specifically mentioned<br>**-Blood loss management strategies:** Not specifically discussed<br>**-Postoperative pain management:** Not detailed<br>**-Mobilization protocols:** Not mentioned explicitly<br><br>**–Outcome Assessment:**<br>**-Definition and measurement of primary outcomes:**<br>● The following outcome was defined:<br>○ **Clinical failure:** An unplanned reoperation during the study period for instrumentation failure or wound-

	related complications (including loosening or breakage of instrumentation, symptomatic pseudarthrosis, wound breakdown at the caudal end of the incision over the pelvic instrumentation, or removal of pelvic fixation because of pain due to hardware prominence). The revision operations performed for proximal junctional kyphosis (PJK) were excluded as the authors considered them a sequela and part of the natural history of long-segment fusions and not a failure of the initial operation.<br>● The measurement method of the outcome was not reported.<br>**-The mean follow-up** was 22 months (median 23 months, range 2–41 months).<br>**-Use of patient-reported outcome measures:** ODI was used to assess undetermined outcome(s) at baseline and at the most recent clinic visit postoperatively<br>**-Radiographic assessment methods:** Not specified<br><br>**–Study Design and Methodology:**<br>-Non-randomized retrospective cohort study with no blinding procedures reported<br>**-Sample size and power calculations:** Not specified<br><br>**–Time-related Factors:**<br>**-Year of study conduction:** The study reviewed cases between December 2009 and March 2012, with follow-up data until November 2013.<br>**-Changes in surgical techniques or technology over time:** Not explored<br><br>**–Funding and Conflicts of Interest:**<br>**-Source of funding:** Not specified<br>**-Declared conflicts of interest:** One of the authors declared being a consultant and stockholder in companies including Amedica, DePuy Synthes, and Medtronic, whereas another declared consultancy for Biomet and AONA.
Elder BD et al., 2017 [34]	**–Patient-Related Factors:**<br>**-Mean Age:** 62 ± 9.8 years (S2AI), 59.2 ± 16 years (IS)<br>**-Sex Ratio:** 63.1% female (S2AI), 56% female (IS)<br>**-BMI:** 30.7 ± 7.2 (S2AI), 30.9 ± 7 (IS)<br>**-Bone quality:** Not specified<br>**-Comorbidities:** Statistically similar rates of diabetes mellitus [18.5% (S2AI), 25% (IS)], osteoporosis (20% in both groups), and osteopenia [12.3% (S2AI), 8% (IS)]<br>**-Severity and type of spinal deformity:** Statistically similar rates of deformity [75.4% (S2AI), 72% (IS)],

	spondylolisthesis [12.3% (S2AI), 8% (IS)], tumor [6.2% (S2AI), 20% (IS)], trauma [9.2% (S2AI), 8% (IS)], and infection [1.5% (S2AI), 0% (IS)] with undefined severity<br>**-Previous spinal surgeries:** Statistically similar rates of prior history of lumbosacral surgery [63.2% (S2AI), 76% (IS)]<br><br>**–Intervention-Related Factors:**<br>**-Laterality:** Not reported<br>**-Surgical screw insertion technique:**<br>● **S2AI:**<br>○ **Starting point:** 20-30 mm caudal to the superior endplate of S1 and 20-25 mm lateral to the midline (patient-specific based on bony anatomy)<br>○ **Exposure:** Bony surface area surrounding the starting point<br>○ **Screw direction:** Towards the greater trochanter, angled 40° laterally and 40° caudally<br>○ **Guidance:** Freehand technique, based on the preoperative computed tomography (CT)<br>○ **Screw size:** Majority 8 or 9 mm in diameter (range 7-10 mm) and 80 or 90 mm in length (range 60-100 mm)<br>○ Trajectory determined based on the preoperative CT<br>● **IS:**<br>○ **Starting point:** PSIS<br>○ **Exposure:** PSIS exposed, recess created in the ilium using a rongeur to minimize screw head prominence<br>○ **Screw direction:** Angled 20-45 degrees caudal and 30-45 degrees lateral<br>○ **Guidance:** Probe-assisted for trajectory determination<br>○ **Screw size:** Majority 8 or 9 mm in diameter (range 7-10 mm) and 80 or 90 mm in length (range 60-100 mm)<br>○ Trajectory determined using a probe<br>● **For both techniques:**<br>○ Screw diameter and length determined based on preoperative CTs<br>○ Majority of patients received 8 or 9 mm (89%) diameter and 80 or 90 mm (75%) length screws<br>**-Use of additional fixation methods:** BMP-2 [51.5% (S2AI), 40% (IS)] used based on the specific criteria (if the patient satisfied two of the 5 conditions: a) prior pseudarthrosis, b) osteoporosis or osteopenia, c) significant focal osteotomy such as pedicle subtraction osteotomy, d) end-stage renal disease, e) smoking, and/or f) elderly)<br>**-Intraoperative imaging techniques:** Not reported<br>**-Use of navigation systems:** Not specified

	**–Surgeon and Hospital-Related Factors:**<br>**-Surgeon's experience:** Not specified<br>**-Surgical volume of the hospital:** Not provided<br>**-Type of hospital (academic vs. community):** Not reported<br>**-Geographical location:** Not mentioned<br><br>**–Perioperative Management:**<br>**-Anesthesia protocols:** Not specifically mentioned<br>**-Blood loss management strategies:** Not specifically discussed<br>**-Postoperative pain management:** Not detailed<br>**-Mobilization protocols:** Not mentioned explicitly<br><br>**–Outcome Assessment:**<br>**-Definition and measurement of primary outcomes:**<br>● The following outcomes were defined:<br>○ **Reoperation:** Any unplanned procedure required for the treatment of pseudarthrosis, device failure, surgical site infection (SSI), wound dehiscence, or device prominence<br>○ **Proximal junctional failure (PJF):** Proximal junctional sagittal Cobb angle between the lower endplate of the uppermost instrumented vertebra (UIV) and the upper endplate of the two supra-adjacent vertebrae ≥ 10° and at least 10° greater than the preoperative measurement<br>○ **Distal device failure:** Failure of devices in the L4-pelvis regions<br>○ **Major pelvic screw loosening:** Clinically symptomatic screw loosening detected on plain X-rays<br>○ **Symptomatic pelvic screw prominence:** Tenderness and/or wound dehiscence in the buttocks immediately overlying the pelvic screw heads<br>○ Back pain was measured by Visual Analogue Scale (VAS) scores.<br>○ Ambulatory status was measured via a 4-point scale (4: Independently ambulatory; 3: Requiring a cane; 2: Requiring a walker; and 1: Wheelchair-bound)<br>● Operative time, expected blood loss, length of Intensive Care Unit (ICU) stay, length of hospital stay, L5-S1 pseudarthrosis, SSI, wound dehiscence, SIJ pain, pelvic fracture, incidental durotomy, nerve injury, major vessel injury, pelvic screw breach, pulmonary embolism, deep vein thrombosis, cardiac complication, and ileus were not defined.

	● SIJ pain was diagnosed by performing the sacral compression test, thigh thrust test, and/or Patrick's test with two positives in three tests required. The measurement method of none of the other outcomes was reported.<br>**-The mean follow-up** was 21.45 months.<br>**-Use of patient-reported outcome measures:** VAS for back pain and ambulatory status, which was rated on the previously mentioned 4-point scale with the assessment made both: preoperatively and at the last follow-up.<br>**-Radiographic assessment methods:** Anterior-posterior and lateral lumbar spine X-rays, and in cases where the fusion status was unclear, computed tomography scans and/or flexion-extension dynamic radiographs were obtained to assess fusion status<br><br>**–Study Design and Methodology:**<br>-The study was a retrospective comparative study<br>-Non-randomized study with no blinding procedures<br>**-Sample size and power calculations:** Not specified<br><br>**–Time-related Factors:**<br>**-Year of study conduction:** The study reviewed cases between October 2010 and December 2014.<br>**-Changes in surgical techniques or technology over time:** Not explored<br><br>**–Funding and Conflicts of Interest:**<br>**-Source of funding:** Gordon and Marilyn Macklin Foundation<br>**-Declared conflicts of interest:** Not reported
Ishida W et al., 2017 [33]	**–Patient-Related Factors:**<br>**-Mean Age:** 61.5 ± 10.7 years (S2AI), 64.3 ± 11.4 years for (IS)<br>**-Sex Ratio:** 67.4% female (S2AI), 70.6% (IS)<br>**-BMI:** 30.2 ± 6.9 (S2AI), 33.0 ± 7.3 (IS)<br>**-Bone quality:** Not specified<br>**-Comorbidities:** Osteoporosis [10.9% (S2AI), 5.9% (IS)]; Osteopenia [23.9% (S2AI), 23.5% (IS)]<br>-Severity and type of spinal deformity: Degenerative scoliosis [56.5% (S2AI), 64.7% (IS)]; Adult idiopathic scoliosis [26.1% (S2AI), 17.6% (IS)]; and Combination [17.4% (S2AI), 17.6% (IS)] with undetermined severity<br>**-Previous spinal surgeries:** Previous lumbosacral surgery [84.8% (S2AI), 82.4% (IS)]; Previous lumbosacral fusion [26.1% (S2AI), 41.2% (IS)]

	**–Intervention-Related Factors:**<br>**-Laterality:** Uni-vs-bilaterality not reported<br>**-Surgical screw insertion technique:** Not detailed but referenced to a previous publication<br>**-Use of additional fixation methods:** BMP-2 [56.5% (S2AI), 41.2% (IS)] used off-label for posterolateral fusion based on an unreported criteria; Cement vertebroplasty at the UIV and/or the supra-adjacent level of UIV [41.3% (S2AI), 41.2% (IS)] used based on surgeons' preference (following an unreported criteria)<br>**-Intraoperative imaging techniques:** Not reported<br>**-Use of navigation systems:** Not specified<br><br>**–Surgeon and Hospital-Related Factors:**<br>**-Surgeon's experience:** Not specified<br>**-Surgical volume of the hospital:** Not provided<br>**-Type of hospital (academic vs. community):** Not reported<br>**-Geographical location:** Not mentioned<br><br>**–Perioperative Management:**<br>**-Anesthesia protocols:** Not mentioned<br>**-Blood loss management strategies:** Not specified<br>**-Postoperative pain management:** Not reported<br>**-Mobilization protocols:** Not specified<br><br>**–Outcome Assessment:**<br>**-Definition and measurement of outcomes:**<br>● The following outcomes were defined:<br>○ **Reoperation rate:** Any unexpected surgery for the treatment of PJK and/or PJF, pseudarthrosis, device failure, SSI, or wound dehiscence<br>○ **PJK:** Proximal junctional sagittal Cobb angle between the lower endplate of the UIV and the upper endplate of the two supra-adjacent vertebrae ≥ 10° and at least 10° greater than the preoperative measurement<br>○ **Distal device failure:** Fractures or pull-out of devices in the L4-pelvis regions<br>○ **Lumbar lordosis (LL):** The angle formed between the superior end plate of L1 and the superior endplate of S1

	○ **Thoracic kyphosis:** The angle formed between the superior end plate of T5 and the inferior end plate of T12<br>○ **SVA:** The distance from the posterosuperior edge of the S1 to the C7 plumb-line<br>○ Back pain was measured by VAS scores.<br>○ Ambulatory status was measured via the previously mentioned 4-point scale.<br>● Operative time, expected blood loss, length of ICU stay, length of hospital stay, PJF, L5-S1 pseudarthrosis, SSI, wound dehiscence, incidental durotomy, Cobb angle, pelvic tilt, sacral slope, pelvic incidence and pelvic incidence–lumbar lordosis (PI-LL) mismatch were not defined.<br>● The radiological parameters were measured in an unreported manner by 2 independent observers. The measurement method of none of the other outcomes was reported, either.<br>**-Mean follow-up** was 21.1 months<br>**-Use of patient-reported outcome measures:** VAS for back pain and ambulatory status, which was rated on the previously mentioned 4-point scale assessed both: preoperatively and at the last follow-up.<br>**-Radiographic assessment methods:** Pre-and-postoperative standing radiographs, and in cases where the fusion status was unclear, computed tomography scans and/or flexion-extension dynamic radiographs were also obtained to assess fusion status and spinopelvic parameters<br><br>**–Study Design and Methodology:**<br>**-Study Design:** Non-randomized retrospective cohort study<br>**-Blinding procedure:** Calculation of radiographic parameters by blinded independent observers at the time of initial presentation and at the last follow-up<br>**-Sample size and power calculations:** Not provided<br><br>**–Time-related Factors:**<br>**-Year of study conduction:** Between October 2010 and February 2015<br>**-Changes in surgical techniques or technology over time:** From October 2010 to December 2013, S2AI screws and traditional IS were equally selected based on surgeons' preference, but from January 2014 to February 2015, S2AI screws were preferentially utilized due to improved clinical outcomes unless there were any contraindications, such as prior history of high sacrectomy or sacral trauma.<br><br>**–Funding and Conflicts of Interest:**<br>**-Source of funding:** Gordon and Marilyn Macklin Foundation<br>**-Declared conflicts of interest:** Several authors disclosed relationships with companies including DePuy Synthes and

	Medtronic.
Nazemi AK et al., 2018 [31]	–**Patient-Related Factors:**<br>**-Mean Age:** 69 ± 9 years (S2AI), 67 ± 9 years (IS)<br>**-Sex Ratio:** 70% female (S2AI), 79% female (IS)<br>**-BMI:** Not reported<br>**-Bone quality:** Not mentioned<br>**-Comorbidities:** Not specified<br>**-Severity and type of spinal deformity:** De novo scoliosis or kyphosis with undetermined severity<br>**-Previous spinal surgeries:** Not mentioned<br><br>–**Intervention-Related Factors:**<br>**-Laterality:** Unilateral screws<br>**-Surgical screw insertion technique:** Specifics not provided<br>**-Use of additional fixation methods:** Not specified<br>**-Intraoperative imaging techniques:** Not reported<br>**-Use of navigation systems:** Not specified<br><br>–**Surgeon and Hospital-Related Factors:**<br>**-Surgeon's experience:** Not specified<br>**-Surgical volume of the hospital:** Not provided<br>**-Type of hospital (academic vs. community):** Not reported<br>**-Geographical location:** Not specified<br><br>–**Perioperative Management:**<br>**-Anesthesia protocols:** Not mentioned<br>**-Blood loss management strategies:** Not specified<br>**-Postoperative pain management:** Not reported<br>**-Mobilization protocols:** Not mentioned<br><br>–**Outcome Assessment:**<br>**-Definition and measurement of outcomes:**<br>● Reoperation rates, L5-S1 pseudarthrosis, sacral insufficiency fracture, hardware prominence, infection, PJK,

	windshield wipering, hardware fracture, hardware removal, LL, pelvic tilt, pelvic incidence, PI-LL mismatch, SVA, Cobb angle, and trunk shift were not defined.<br>● Clinical notes were reviewed for clinical outcomes and the Surgimap Spine 2.2.10 (Nemaris Inc., New York, NY, USA) software were reviewed for radiological outcomes.<br>● Postoperative QoL measures were defined as back pain and leg pain. They were assessed via VAS and ODI scores.<br>**-Mean follow-up** was 27 ± 17 months<br>**-Use of patient-reported outcome measures:** ODI and VAS for back pain, and VAS for leg pain were adopted over a 2-year postoperative period.<br>**-Radiographic assessment methods:** Postoperative lumbar posterioanterior and lateral films (1.5 and 3 months) and full-length scoliosis standing radiographs (0.5 and 1 year) were adopted to assess the above mentioned radiological parameters.<br><br>**–Study Design and Methodology:**<br>**-Study Design:** Non-randomized retrospective cohort study<br>-No specific blinding procedures were mentioned.<br>**-Sample size and power calculations:** Not provided<br><br>**–Time-related Factors:**<br>**-Year of study conduction:** Not explicitly stated, but the study was published in 2018 with patients followed for an average of 27 months<br>**-Changes in surgical techniques or technology over time:** Not explored<br><br>**–Funding and Conflicts of Interest:**<br>**-Source of funding:** Reported to be none<br>**-Declared conflicts of interest:** Declared to be none

BMI = Body mass index; S2AI = S2 Alar-iliac; IS = (Traditional) iliac screws; SVA = Sagittal vertical axis; HRQoL = Health-related quality of life; ODI = Oswestry disability index; SF-36; PCS and MCS = 36-item short-form physical component and mental component health surveys; SRS-22 = Scoliosis Research Society 22-item patient questionnaire; SIJ = Sacroiliac joint; PSIS =

Posterior superior iliac spine; BMP = Bone morphogenetic protein; PJK = Proximal junctional kyphosis; CT = Computed tomography; SSI = Surgical site infection; PJF = Proximal junctional failure; UIV = Uppermost instrumented vertebra; VAS = Visual Analogue Scale; ICU = Intensive care unit; LL = Lumbar lordosis; PI-LL = Pelvic incidence–lumbar lordosis.
